# Dysfunction of programmed embryo senescence is linked to genetic developmental defects

**DOI:** 10.1242/dev.200903

**Published:** 2023-05-03

**Authors:** Cristina de Lope, Rebeca García-Lucena, Marta Magariños, Yolanda León, Nuria Casa-Rodríguez, Nuria Contreras, Carmen Escudero-Iriarte, Isabel Varela-Nieto, Pascal Maire, Ignacio Palmero

**Affiliations:** ^1^ Cell Senescence and Tumor Suppression Lab, Instituto de Investigaciones Biomédicas “Alberto Sols” CSIC-UAM, 28029 Madrid, Spain; ^2^ Biology Department, Universidad Autónoma de Madrid, 28049 Madrid, Spain; ^3^ Rare Diseases Networking Biomedical Research Centre (CIBERER), CIBER, Carlos III Institute of Health, 28029 Madrid, Spain; ^4^ Neuropathology of Hearing and Myelinopathies Lab, Instituto de Investigaciones Biomédicas “Alberto Sols” CSIC-UAM, 28029 Madrid, Spain; ^5^ Hospital La Paz Institute for Health Research (IdiPAZ), 28046 Madrid, Spain; ^6^ Université de Paris Cité, Institut Cochin, INSERM, CNRS, 75014 Paris, France

**Keywords:** Cellular senescence, Inner ear, SIX1, BOR syndrome

## Abstract

Developmental senescence is a form of programmed senescence that contributes to morphogenesis during embryonic development. We showed recently that the SIX1 homeoprotein, an essential regulator of organogenesis, is also a repressor of adult cellular senescence. Alterations in the SIX/EYA pathway are linked to the human branchio-oto-renal (BOR) syndrome, a rare congenital disorder associated with defects in the ears, kidneys and branchial arches. Here, we have used *Six1*-deficient mice, an animal model of the BOR syndrome, to investigate whether dysfunction of senescence underpins the developmental defects associated with SIX1 deficiency. We have focused on the developing inner ear, an organ with physiological developmental senescence that is severely affected in *Six1*-deficient mice and BOR patients. We show aberrant levels and distribution of senescence markers in *Six1*-deficient inner ears concomitant with defective morphogenesis of senescent structures. Transcriptomic analysis and *ex vivo* assays support a link between aberrant senescence and altered morphogenesis in this model, associated with deregulation of the TGFβ/BMP pathway. Our results show that misregulation of embryo senescence may lead to genetic developmental disorders, significantly expanding the connection between senescence and disease.

## Teaser

**Highlighted Article:** Analysis of a mouse model of a human congenital syndrome reveals that misregulation of embryo senescence may contribute to genetic developmental disorders, thus expanding the link between cellular senescence and disease.

## INTRODUCTION

Cellular senescence is a stable form of cell cycle arrest, relevant in diverse physiological and pathological settings (Chan and Narita, 2019; Ou et al., 2020). Senescence can act as a stress response elicited by many forms of cellular damage, including DNA damage or oncogene activation, among others. In parallel, senescence also participates in the control of tissue homeostasis in the context of normal physiology (Muñoz-Espín and Serrano, 2014). In this regard, recent evidence has shown that a form of programmed senescence is active during vertebrate embryonic development (Muñoz-Espín et al., 2013; Storer et al., 2013; Davaapil et al., 2017; Villiard et al., 2017; Gibaja et al., 2019). Developmental senescence occurs transiently in specific embryonic structures, contributing to their clearance or remodeling in a process often linked to apoptosis or macrophage-mediated cell clearance. Senescent cells may also provide paracrine instructive signals required for embryo patterning. This program is evolutionarily conserved and has been described so far in humans, mice, birds, amphibians and fish (Da Silva-Álvarez et al., 2019; Varela-Nieto et al., 2019; Czarkwiani and Yun, 2018). Developmental senescence shares some of the major features of adult senescence, including cell-cycle arrest and a characteristic secretome, but it seems to involve a specific molecular mechanism mediated mainly by the cell-cycle inhibitor p21Cip1 (Cdkn1a), in a p53- and DNA damage-independent manner (Muñoz-Espín et al., 2013; Storer et al., 2013). Dysfunction of the adult senescence program is linked to a large variety of diseases, from cancer to diabetes and neurodegeneration (Muñoz-Espín and Serrano, 2014). However, it is unclear whether altered embryo senescence may similarly contribute to the pathogenesis of congenital diseases. The human branchio-oto-renal (BOR) syndrome is a rare developmental disorder characterized by hearing loss, renal anomalies and defective closure of branchial arches (Knoers and Cremers, 2009). This syndrome is linked to mutations in genes of the SIX/EYA pathway such as *EYA1*, *SIX1 or SIX5* (Ruf et al., 2004; Knoers and Cremers, 2009). SIX proteins are key developmental transcriptional regulators that cooperate with co-factors of the EYA family to control organogenesis (Xu, 2013). In particular, *Six1-*deficient mice show severe developmental defects in the ear and kidney, among other organs, which strongly resemble BOR syndrome symptoms (Laclef et al., 2003b; Zheng et al., 2003; Ozaki et al., 2004; Xu et al., 2003; Bosman et al., 2009). Of note, we have recently shown that the SIX1 homeoprotein can function as an essential negative regulator of adult cell senescence (Adrados et al., 2016; De Lope et al., 2019). With this background, here we set out to study whether the organogenesis defects in *Six1*-deficient mice, considered an animal model of the human developmental BOR syndrome, may be linked to disruption of the physiological program of developmental senescence.

## RESULTS AND DISCUSSION

To investigate the role of cellular senescence in developmental defects associated with a defective SIX/EYA pathway, we focused on the developing inner ear, a specialized neurosensory organ responsible for hearing and balance (Magariños et al., 2012; Varela-Nieto et al., 2019). Physiological developmental senescence occurs in the inner ear, specifically in the endolymphatic duct and sac located in its dorsal end (Muñoz-Espín et al., 2013). In addition, ear morphogenesis and hearing are severely affected in *Six1*-deficient mice (Zheng et al., 2003; Ozaki et al., 2004; Laclef et al., 2003b) and in human BOR patients (Chen et al., 2021).

**Fig. 1.**
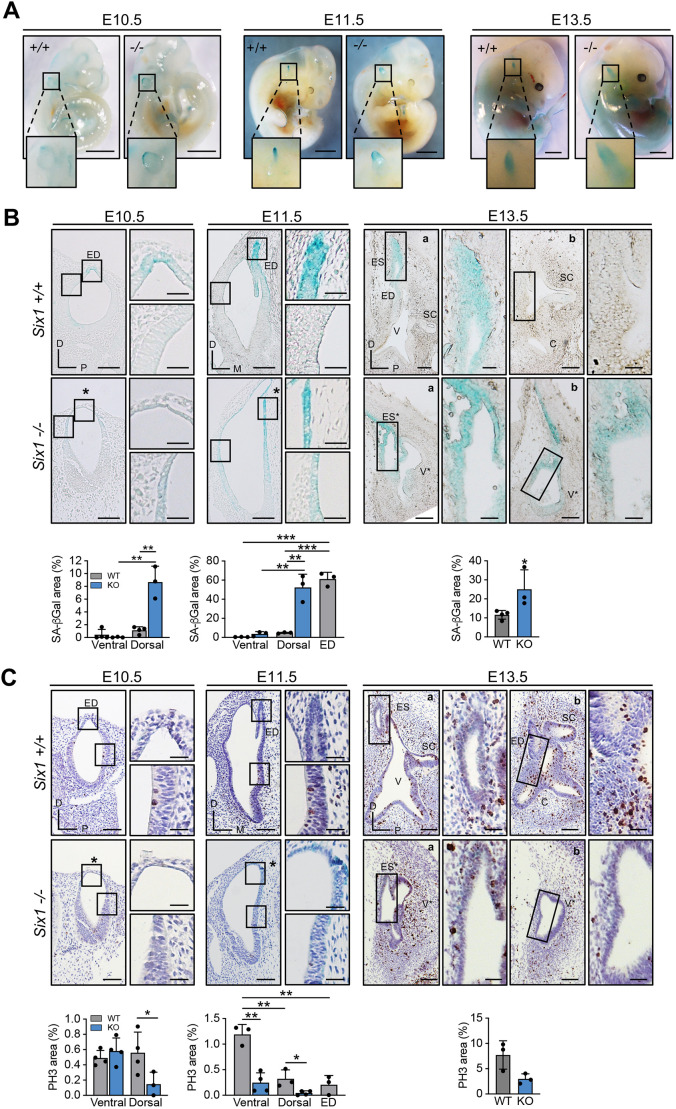
**Aberrant senescence in *Six1*-deficient inner ear.** (A) Representative images of *Six1^+/+^* or *Six1^−/−^* embryos stained for SA-βGal at E10.5, E11.5 and E13.5. Insets show the inner ear at higher magnification. Scale bars: 1 mm. (B) Top: Representative images of SA-βGal staining in sections from *Six1^+/+^* and *Six1^−/−^* inner ears at the indicated stages. E10.5 and E13.5, longitudinal sections. E11.5, transversal sections. Scale bars: 100 µm (main panels); 30 µm (insets). Bottom: Quantification of SA-βGal-positive area. *n*=3 except for E10.5 WT (*n*=4). (C) Representative images of phospho-histone H3 immunohistochemistry (top) and quantification of positive area (bottom) as described in B. E10.5 WT and E11.5 WT and KO, *n*=4; E10.5 KO and E13.5 WT and KO, *n*=3. In E10.5 and E11.5 *Six1^−/−^* inner ears, asterisks identify aberrant dorsal area. In E13.5 *Six1^−/−^* inner ears, ES* and V* identify endolymphatic sac-like and vestibule-like structures. a and b in panels B and C indicate sections along the latero-medial axis of the same embryo. Boxed areas are shown at higher magnification to the right. C, cochlea; D, dorsal; ED, endolymphatic duct; ES, endolymphatic sac; KO, knockout; M, medial; P, posterior; SC, semicircular canals; V, vestibule; WT, wild type. Error bars represent s.d.

We analyzed wild-type and *Six1*-deficient embryos at key developmental stages for the formation of the endolymphatic sac [embryonic day (E) 10.5 to E13.5]. During this time window, normal inner ear development progresses from an initial stage of a largely undifferentiated otic vesicle at E10.5 to a high degree of complexity at E13.5 when the specialized structures of the adult inner ear can be distinguished (Magariños et al., 2012; Varela-Nieto et al., 2019). A dorsal primordium of the endolymphatic duct becomes evident at the dorsal end of the otic vesicles at E10.5 and then elongates dorsally and widens at its distal end to form the endolymphatic sac (Fig. 1A-C). Activity of senescence-associated beta-galactosidase (SA-βGal), a classical senescence marker, was observed in the inner ear and other senescent structures, such as the neural tube, the apical ectodermal ridge and the tip of the tail (Fig. 1A, Fig. S1A), in agreement with previous reports (Muñoz-Espín et al., 2013). In the inner ear, SA-βGal activity was restricted to the endolymphatic sac and duct at E13.5 and to the primordium of these structures at early stages (E10.5 and E11.5; Fig. 1A,B). In agreement with published studies (Zheng et al., 2003; Ozaki et al., 2004), Six1 could be detected by immunohistochemistry in the ventral region of the developing inner ear at early stages, being restricted later to prosensory regions (Fig. S1B,C). *Six1-*deficient developing inner ears displayed severe defects in the morphogenesis of the endolymphatic duct and other structures (Fig. 1A,B), consistent with previous reports (Zheng et al., 2003; Ozaki et al., 2004; Laclef et al., 2003b). At early stages (E10.5 to E11.5), *Six1-*deficient otocysts showed a widened dorsal area without a distinct elongated endolymphatic duct, and also lacked the initial coiling of the cochlea in the ventral region (Fig. 1A,B). At these stages, the SA-βGal-positive area extended ventrally relative to the wild-type pattern, reaching almost the entire epithelium of the inner ear in some cases at E11.5 (Fig. 1A,B). At E13.5, *Six1-*deficient inner ears displayed a grossly abnormal morphology with an enlarged aberrant endolymphatic sac-like structure and an absence of distinct semicircular canals or cochlea (Fig. 1A-C). A clear expansion of the SA-βGal-positive zone was observed in mutant embryos at this stage, which encompassed the aberrant endolymphatic sac and adjacent regions of the inner ear (Fig. 1A,B). To characterize this phenotype further, we also measured the proliferation rate, a hallmark of senescence, using phospho-histone H3 (PH3) staining. At stages E10.5 and E11.5, a significant decrease in proliferation was observed in the aberrant SA-βGal-positive regions of the mutant otic vesicles (Fig. 1C). In E13.5 control embryos, the endolymphatic sac showed reduced proliferation, consistent with its senescent nature (Muñoz-Espín et al., 2013). In *Six1-*deficient embryos, reduced proliferation levels were observed across the malformed inner ear (Fig. 1C), overlapping with aberrant SA-βGal activity. Taken together, these results support the existence of aberrant developmental senescence during morphogenesis of the inner ear in *Six1-*deficient embryos.

**Fig. 2.**
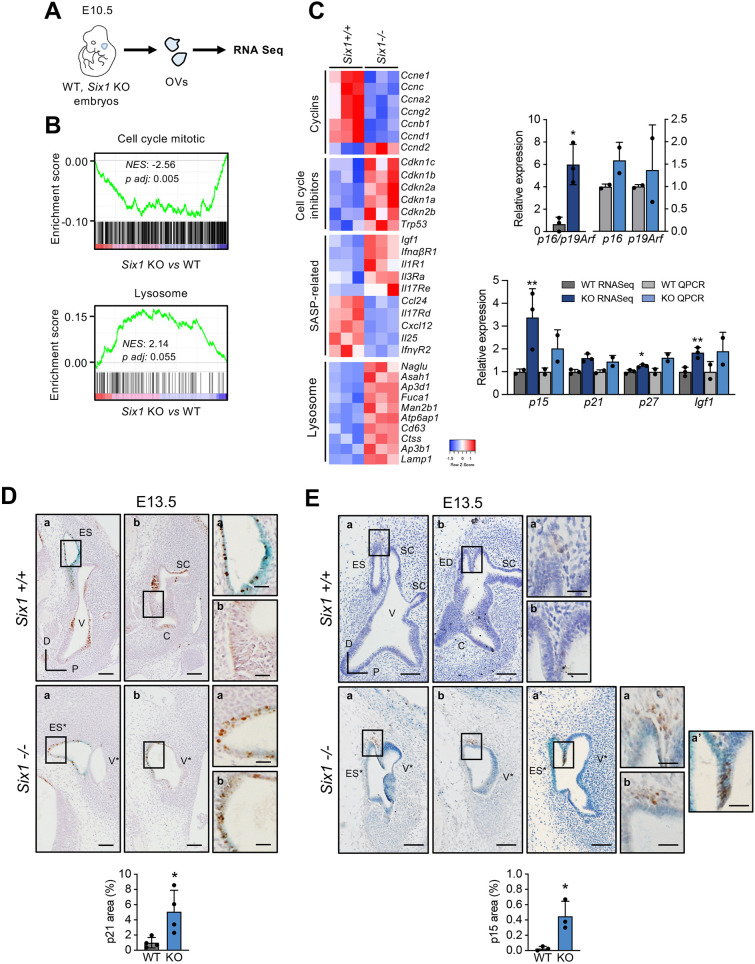
**Senescence markers in *Six1*-deficient inner ear.** (A) Experimental design for the RNA-Seq experiment. OVs, otic vesicles. (B) Enrichment plots for the gene sets ‘Cell_Cycle_Mitotic’ (Reactome) and ‘Lysosome’ (KEGG). NES, normalized enrichment score. (C) Left: Heatmap analysis of the expression of senescence-related genes in *Six1-*deficient (KO) versus wild-type (WT) otic vesicles. SASP, senescence-associated secretory phenotype. Right: RNA-Seq results and QPCR validation of the indicated genes relative to WT samples (WT and KO RNA-Seq *n*=3 pools of 6 vesicles each; WT and KO QPCR *n*=2 pools of 6 vesicles each). (D) Representative images of p21 immunohistochemistry in longitudinal sections of E13.5 *Six1^+/+^* and *Six1^−/−^* inner ear and quantification of senescence-associated p21-positive area (*n*=4 for each genotype). (E) Representative images of p15 immunohistochemistry and p15-positive area quantification (*n*=3 for each genotype) as described in D. Scale bars: 100 µm (main panels); 30 µm (insets). C, cochlea; ED, endolymphatic duct; ES, endolymphatic sac; SC, semicircular canals; V, vestibule. In *Six1^−/−^* inner ears, ES* and V* identify endolymphatic sac-like and vestibule-like structures. a and b in panels D and E indicate sections along the latero-medial axis of the same embryo. a' indicates an equivalent section from a different embryo. Boxed areas are shown at higher magnification to the right. Error bars represent s.d.

To gain insights into the molecular basis of the aberrant senescent phenotype in the inner ear of *Six1-*deficient embryos, we carried out transcriptomic analysis using RNA sequencing (RNA-Seq) in wild-type and mutant otic vesicles at E10.5 (Fig. 2A). Functional enrichment analysis identified differences in senescence-related categories, such as negative enrichment in ‘cell cycle’ or ‘DNA replication’ and positive enrichment in ‘protein secretion’ or ‘lysosomal activity’ categories (Fig. 2B, Table S1). Of note, no significant enrichment was observed for gene sets associated with different forms of adult senescence, in agreement with previous studies (Storer et al., 2013; Muñoz-Espín et al., 2013). A general increase in Cdk inhibitors associated with senescence, such as *p15Ink4b* (*Cdkn2b*), *p21Cip1* (*Cdkn1a*), *p27Kip1* (*Cdkn1b*), *p57Kip2* (*Cdkn1c*) and the *Cdkn2a* locus (*p16Ink4a and p19Arf*), was observed, together with an overall decrease in the expression of cyclins. For a selection of genes of interest, the RNA-Seq results were validated by QPCR, using independent control and *Six1-*deficient samples (Fig. 2C). In agreement with previous reports (Storer et al., 2013; Muñoz-Espín et al., 2013), we did not observe a significant increase in cytokines or chemokines associated with the adult canonical inflammatory SASP (senescence-associated secretory phenotype), such as *Il6*. However, an increase was observed for receptors such as *Il3ra*, or *Il1r1*, together with a decrease in factors such as *Il25*, *Ccl24* and *Cxcl12* (Fig. 2C). Notably, we also found increased levels of *Igf1*, which has recently been linked to ear senescence in chicken (Gibaja et al., 2019). Collectively, these gene expression results are consistent with the existence of aberrant senescence in the inner ear of *Six1*-deficient embryos.

Next, we complemented this transcriptomic analysis with evaluation of key senescence effectors by immunohistochemistry. p21Cip1 has been proposed as a major mediator of developmental senescence in the inner ear and other embryo structures (Storer et al., 2013; Muñoz-Espín et al., 2013). p21 staining could be detected in wild-type embryos from E11.5 onwards in pro-neurosensory domains (Fig. S1D), as previously described (Mantela et al., 2005). However, senescence-associated p21 staining could only be detected at E13.5 in the endolymphatic duct and sac, colocalizing with SA-βGal (Fig. 2D; Muñoz-Espín et al., 2013). In E13.5 *Six1-*deficient embryos, p21 staining in the aberrant SA-βGal-positive otic epithelium was elevated, resulting in a global increase in senescence-associated p21 staining. (Fig. 2D). p15Ink4b has also been linked to mouse developmental senescence in several organs (Storer et al., 2013; Muñoz-Espín et al., 2013). In wild-type embryos, p15 positivity was detected at stage E13.5 in the mesenchyme surrounding the endolymphatic sac (Fig. 2E), as previously described (Muñoz-Espín et al., 2013). In *Six1*-deficient embryos, an increase in p15 positivity was detected in the mesenchyme surrounding the areas with aberrant senescence. Positive cells were also observed in some cases in defined areas of the mutant inner ear epithelium. Quantification of the p15-positive area indicated a significant increase in the SA-βGal positive epithelium of *Six1*-deficient embryos relative to the equivalent region in wild-type embryos (Fig. 2E).

**Fig. 3.**
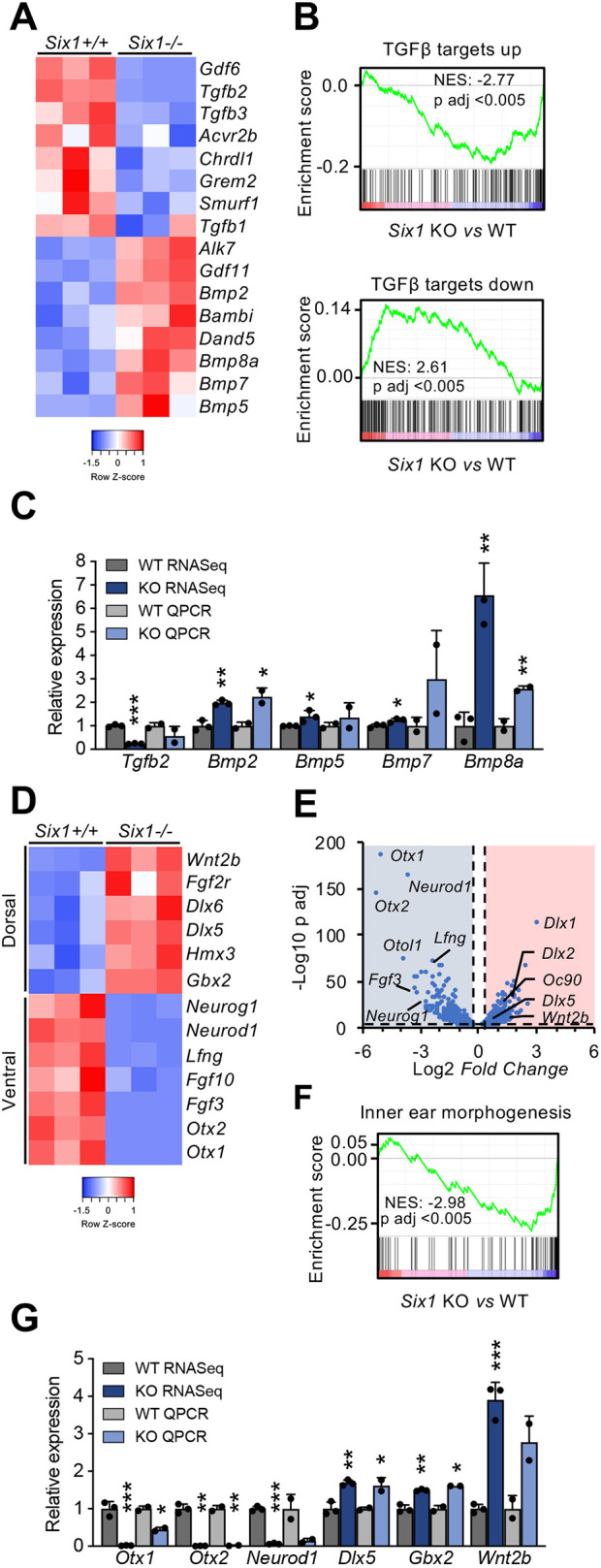
**TGFβ pathway and dorso-ventral axis alteration in *Six1*-deficient otic vesicles.** (A) Heatmap analysis of the expression of genes of the TGFβ pathway in *Six1^−/−^* (KO) versus *Six1^+/+^* (WT) otic vesicles. *Alk7*, *Acvr1c*. (B) Enrichment plots for the gene sets ‘Plasari_Tgfβ_Targets_10h_up’ and ‘Plasari_Tgfβ_Targets_10h_down’ (Chemical and Genetic Perturbations). NES, normalized enrichment score. (C) RNA-Seq results and QPCR validation of the indicated genes in *Six1^−/−^* versus wild-type (WT and KO RNA-Seq, *n*=3 pools of 6 otic vesicles each; WT and KO QPCR *n*=2 pools of 6 otic vesicles each). (D) Heatmap analysis of ventral and dorsal markers, as described in A. (E) Volcano plot analysis showing some of the most downregulated genes (blue shaded area) and upregulated genes (red shaded area) in the RNA-Seq analysis. The vertical dashed lines indicate 1.2- or −1.2-fold change and horizontal dashed line indicates *P*=0.05. (F) Enrichment plot for the gene set ‘Inner_Ear_Morphogenesis’ (Gene Ontology). (G) RNA-Seq results and QPCR validation of ventral and dorsal genes, as described in C. Error bars represent s.d.

We also analyzed the RNA-Seq results to identify potential mechanisms involved in the phenotype observed in *Six1-*deficient inner ears. Gene set enrichment analysis revealed alterations in *Six1*-defective embryos of the TGFβ, Notch and PI3K/mTOR signaling pathways (Fig. 3A-C, Fig. S2A,B, Table S1). The TGFβ pathway plays an essential role in morphogenesis of the inner ear and other organs during embryonic development (Jia and Meng, 2021; Ma et al., 2019; David and Massagué, 2018). It has also been implicated in diverse senescence settings, including developmental senescence in the inner ear and other structures (Muñoz-Espín et al., 2013; Gibaja et al., 2019; Davaapil et al., 2017; Varela-Nieto et al., 2019). Moreover, TGFβ has also been previously connected to SIX1 in the context of cancer progression (Micalizzi et al., 2010). *Six1-*deficient otic vesicles showed a global deregulation of the pathway, with reduced expression of members of the TGFβ branch (such as *Tgfb2* and *Tgfb3*) and increased expression of BMP factors (such as *Bmp2*, *Bmp5, Bmp7* and *Bmp8a*) (Fig. 3A-C). Of note, RNA-Seq also revealed a marked deregulation in *Six1*-deficient vesicles of the gene program responsible for the establishment of the dorso-ventral pattern in developing inner ears (Ohta and Schoenwolf, 2018), with increased dorsal markers and downregulated ventral markers. *Wnt2b*, a marker of the endolymphatic duct and sac (Sienknecht and Fekete, 2009), was also significantly increased, consistent with the morphological changes in mutant inner ears (Fig. 3D-G). This differential expression pattern is consistent with a dorsalization phenotype in *Six1-*deficient otic vesicles, as suggested in previous studies (Ozaki et al., 2004), and it may suggest that dorso-ventral patterning and senescence regulation are inter-related processes in the inner ear and perhaps other developmental structures.

**Fig. 4.**
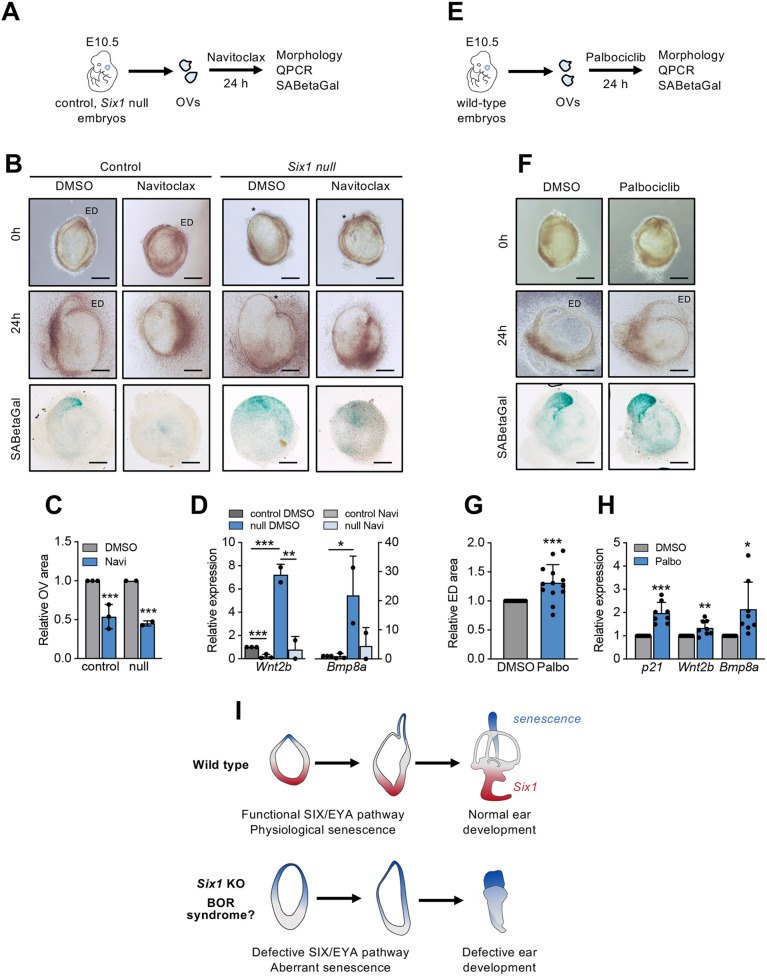
**Senescence manipulation in otic vesicles *ex vivo.*** (A) Scheme of the experimental design for navitoclax treatment *ex vivo*. (B) Representative images of E10.5 control (wild type and heterozygous) or *Six1-*deficient otic vesicles treated with 2 µM navitoclax or DMSO at 0 and 24 h and after SA-βGal staining. In *Six1-*deficient, asterisks mark aberrant dorsal areas. Scale bars: 200 µm. (C) Quantification of the otic vesicle area after 24 h treatment with navitoclax (*n*=3 independent assays with at least 6 vesicles per genotype). (D) QPCR analysis of *Wnt2b* and *Bmp8a* expression in control and *Six1-*deficient otic vesicles treated with Navitoclax or DMSO (*n*=3 independent assays). (E) Scheme of the experimental design for palbociclib treatment *ex vivo*. (F) Representative images of E10.5 wild-type otic vesicles treated with 2 µM palbociclib or DMSO at 0 and 24 h and after SA-βGal staining. Scale bars: 200 µm. (G) Quantification of the endolymphatic duct area after 24 h treatment with palbociclib (*n*=5 independent assays with 13 vesicles). (H) QPCR analysis in wild-type otic vesicles treated with palbociclib (*n*=8 independent assays). (I) Summary of the proposed model of aberrant senescence in *Six1-*deficient inner ears. ED, endolymphatic duct; OVs, otic vesicles. Error bars represent s.d.

To characterize further the aberrant senescent phenotype in *Six1-*deficient developing inner ear and its link to defective morphogenesis, we performed experiments with otic vesicles *ex vivo* (Fig. 4A,E; León et al., 1995). E10.5 *Six1-*deficient and control vesicles essentially retained their characteristic morphology (Fig. 4B, Fig. S1E) under *ex vivo* conditions, with a differentiated SA-βGal-positive endolymphatic duct in controls and an aberrant SA-βGal-positive dorsal region in *Six1-*deficient vesicles. Treatment with the senolytic drug navitoclax in control vesicles led to reduced size or absence of a distinct endolymphatic duct, consistent with the senescent nature of this structure (Fig. 4B,C, Fig. S3A). In *Six1-*deficient vesicles, the distinctive enlarged SA-βGal-positive dorsal region was no longer distinguishable after navitoclax treatment, further supporting its link to dysregulated senescence (Fig. 4B,C, Fig. S3A,C). As expected (see Fig. 3C,G), transcripts for the endolymphatic sac marker *Wnt2b* and for *Bmp8a*, one of the most upregulated Bmp factors, were significantly elevated in untreated *Six1*-deficient *ex vivo* vesicles (Fig. 4D, Fig. S1F). Interestingly, navitoclax caused a clear reduction of both transcripts, suggesting that their upregulation in *Six1*-deficient inner ear is associated with aberrant senescence. In a reverse approach, we induced senescence in wild-type otic vesicles with the senogenic agent palbociclib. Treatment with palbociclib led to morphological changes reminiscent of the *Six1*-deficient phenotype, with a significant enlargement of the SA-βGal-positive endolymphatic sac area and increased expression of *Wnt2b* (Fig. 4F-H, Fig. S3B,D). The *Bmp8a* transcript was also significantly increased in palbociclib-treated vesicles, further supporting its link to senescence in our model (Fig. 4H). An increase in *p21Cip1* was also observed with palbociclib, serving as an internal control of the effectiveness of the treatment. Of note, inhibition of BMP signaling with dorsomorphin partially reverted the aberrant expression of key genes in *Six1*-deficient vesicles (Fig. S3E). Taken together, these experiments support the existence of aberrant senescence linked to BMP deregulation and morphological defects in the developing inner ear of *Six1*-deficient embryos.

In summary, we show here that aberrant programmed senescence is associated with defective morphogenesis in the developing inner ear of *Six1*-deficient mice, an animal model of the human developmental BOR syndrome. Our observations suggest that, in parallel to its well-established function in patterning and morphogenesis, Six1 may act as a physiological repressor of senescence during inner ear development, contributing to delineation of the senescent domain in this organ. However, we cannot formally exclude the possibility that *Six1* deficiency may lead to ectopic embryo senescence via alternative mechanisms. Our data also support that aberrant senescence in *Six1*-deficient ears could be mediated, at least in part by a paracrine process involving BMP deregulation. This mechanism would differ from TGFβ-mediated physiological developmental senescence (Gibaja et al., 2019; Muñoz-Espín et al., 2013; Davaapil et al., 2017), but would be consistent with the known pro-senescence role of BMP signaling in different settings (Kaneda et al., 2011; Acosta et al., 2013), including embryonic development (Storer et al., 2013). Further studies will be necessary for an in-depth understanding of the impact of senescence misregulation in Six1-associated developmental defects and the mechanisms involved, including the potential genetic rescue of these phenotypes by a deficiency in candidate senescence mediators, such as the products of the *Cdkn1a* or *Cdkn2a* loci. Finally, our results may have important implications regarding the general role of senescence in disease (Fig. 4I). Extensive evidence indicates that senescence dysfunction in the adult contributes to the pathogenesis of a large number of diseases (Paez-Ribes et al., 2019). Also, recent results suggest that maternally or drug-induced embryo defects may be linked to senescence (Xu et al., 2021; Rhinn et al., 2022). To our knowledge, our data is the first evidence of aberrant senescence in an animal model of a human genetic developmental syndrome. Thus, our study significantly expands the link of senescence to disease, indicating that alterations in the physiological program of developmental senescence may contribute to the pathogenesis of congenital genetic defects.

## MATERIALS AND METHODS

### Mouse strains

*Six1*-*lacZ* (Six1^tm1Mair^; Laclef et al., 2003a) and *Six1*-*loxP* (Six1^tm2.1Mair^; Le Grand et al., 2012) mice were used in a C57BL/6 background. *Six1*-cKO mice, lacking a *lacZ* cassette, were generated by crossing *Six1*-*loxP* mice with ubiquitous Cre-expressor CAG-Cre mice [Tg(CAG-cre)1Nagy; Belteki et al., 2005] provided by Andras Nagy and Marina Gertsenstein (Lunenfeld-Tanenbaum Research Institute, Toronto, Canada) (Fig. S1G). *Six1*-cKO mice were used for immunohistochemistry and SA-βGal assays in embryos or *ex vivo* vesicles. *Six1*-*lacZ* mice were used for RNA-Seq experiments and other *ex vivo* assays. Indistinguishable results were obtained with both strains, which are collectively described in the text as *Six1* deficient. Both female and male embryos were used with undistinguishable results. All animal experiments were performed in accordance with regional, national and EU guidelines and regulations, approved by the ethics committees of the Instituto de Investigaciones Biomédicas and the Spanish Research Council (CSIC), and authorized by the Madrid Regional Government.

### SA-βGal staining

Mouse embryos at stages E10.5, E11.5 and E13.5 were extracted and fixed in 2% formaldehyde, 0.2% glutaraldehyde in PBS for 45 min with shaking at room temperature, washed in PBS and incubated with an X-Gal staining solution at pH 5.5 as described by Dimri et al. (1995), for 4-5 h at 37°C with shaking and protected from light. After incubation, embryos were washed in PBS, dehydrated with isopropanol to preserve βGal staining and embedded in paraffin for serial sectioning. For SA-βGal staining, 5 µm sections were dewaxed and rehydrated in a 100%, 95% and 70% ethanol series, washed in PBS and mounted without any counterstain. For *ex vivo* otic vesicles, these were fixed in the same fixation solution for 15 min at room temperature, washed in PBS and incubated with the staining solution at pH 6 overnight at 37°C, protected from light. Images were acquired with a Nikon 90i microscope. Quantification was performed using the Fiji package of ImageJ software (National Institutes of Health, Bethesda, Maryland, USA). Regions of interest including the epithelium of otic vesicles were created using the ‘Freehand’ tool. Color deconvolution was performed using the Giemsa option and the ‘Threshold’ tool was used to define positive and negative areas. At least three different sections per embryo (corresponding to the region of the vesicle including the endolymphatic sac in wild-type embryos) were used for quantification.

### Immunohistochemistry in paraffin sections

For immunohistochemistry, serial 5 µm sections from SA-βGal-stained embryos embedded in paraffin as detailed above were processed as described by De Lope et al. (2019) using the primary antibodies shown in Table S2. Immunohistochemistry images were obtained with a Nikon 90i microscope and quantification of positive areas was performed with Fiji software as described above, using the ‘H DAB’ option for color deconvolution.

### RNA-Seq

Otic vesicles from wild-type or *Six1*^−/−^ embryos at stage E10.5 were dissected with sharpened tweezers and frozen in lysis buffer from the RNeasy Micro Kit (QIAGEN). For RNA-Seq, the otic vesicles were pooled in three sets for each genotype, each pool containing six vesicles from three embryos (in total, 18 vesicles per genotype). Total RNA was extracted with the RNeasy Micro Kit according to the manufacturer's instructions, followed by DNase treatment using the Turbo DNA-free kit (Thermo Fisher Scientific). For sequencing, 10-30 ng of total RNA was used for each sample. The average RNA integrity number was 9.4 (range 9-9.6), as measured on an Agilent 2100 Bioanalyzer. Sequencing libraries were prepared with the QuantSeq 3′ mRNA-Seq Library Prep Kit (FWD) for Illumina (Lexogen) following the manufacturer's instructions. Libraries were completed by PCR, applied to an Illumina flow cell for cluster generation and sequenced on an Illumina HiSeq 2500 with v4 Chemistry, following manufacturer's protocols. Image analysis, per-cycle base-calling and quality score assignment was performed with Illumina HiSeq Control Software. Conversion of BCL files to FASTQ format was performed with bcl2fastq software (Illumina). Differential gene expression analysis was performed with DESeq2 within Bluebee (Lexogen QuantSeq DE) using the GRCm38 mouse genome build. The functional enrichment analysis was performed using the Gene Set Enrichment Analysis tool (GSEA, Broad Institute, USA) to identify genes with statistically significant differential expression (*P*adj≤0.05) using either the overlap mode for overexpressed or repressed genes or the pre-ranked mode for all genes ranked with the formula (−log10*P*adj×log2FC).

### Quantitative PCR

Total RNA was obtained from otic vesicles freshly removed from embryos or after *ex vivo* assays as described above. Quantitative real-time PCR was performed essentially as described by Gómez-Cabello et al. (2010). Primer sequences are described in Table S3.

### *Ex vivo* culture of otic vesicles

Otic vesicles were dissected from E10.5 mouse embryos with sharpened tweezers, washed in serum-free Dulbecco's Modified Eagle Medium (DMEM, GIBCO), transferred to 24-well plates and treated with 2 µM palbociclib (MedChem Express), 2 µM navitoclax (MedChem Express) or 2 µM dorsomorphin (Abcam) in DMEM (10% fetal bovine serum, 1% for dorsomorphin) containing 0.5% antibiotics (penicillin and streptomycin), for 24 h at 37°C in 5% CO_2_. The same volume of solvent was used as control in each case. Images of the *ex vivo* cultured otic vesicles were taken at time 0 and after 24 h and total RNA was extracted from vesicles for quantitative real-time PCR as described above. For navitoclax experiments, the control group included both wild-type and heterozygous embryos. Quantification of the total otic vesicle area, endolymphatic sac area or SA-βGal-positive area was performed with Fiji software as described above.

### Statistical analysis

Statistical significance was calculated using unpaired, two-tailed Student's *t*-tests (***P<0.001; **P<0.01; *P<0.05).

## Supplementary Material

10.1242/develop.200903_sup1 Supplementary information
