# Supplementary material for: Dysfunction of programmed embryo senescence is linked to genetic developmental defects
Source: Development. 2023 May 3;150(9):dev200903. doi: 10.1242/dev.200903 (PMC10259514; doi:10.1242/dev.200903)
Supplement: Supplementary information [file develop-150-200903-s1.pdf]

Figure S1

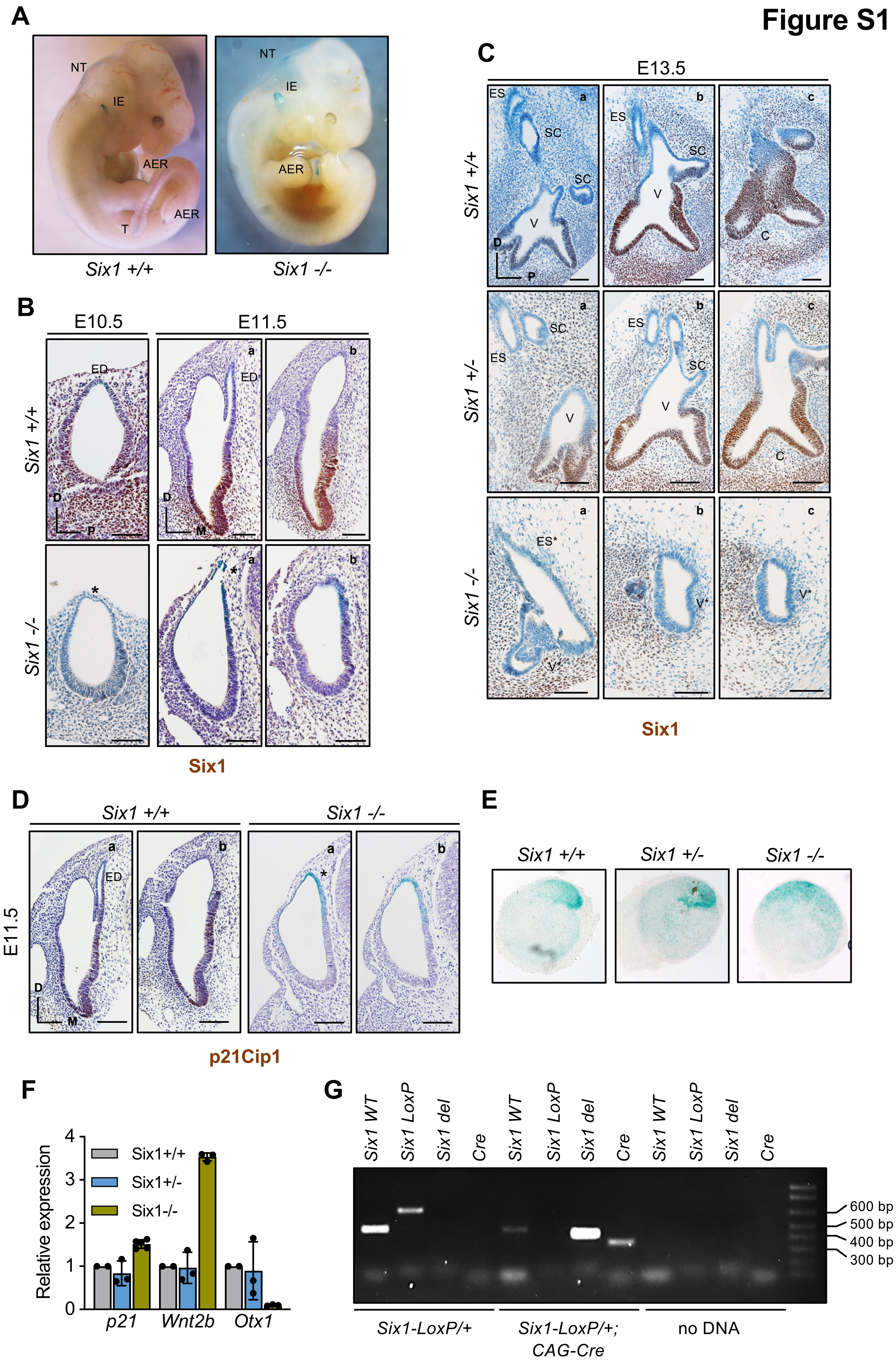

Development • Supplementary information

Fig. S1. (A) Representative image of a *Six1+/+* and *Six1-/-* embryos stained for SA-BetaGal at E10.5. (B, C) Representative images of Six1 immunohistochemistry in longitudinal (E10.5 and E13.5) or transversal (E11.5) sections from *Six1 +/+*, *Six1 +/-* and *Six1 -/-* inner ears. (D) Representative images of p21 immunohistochemistry in transversal sections from embryos of the indicated genotypes at stage E11.5. Scale bars, 100 µm. NT, neural tube, IE, inner ear, AER, apical ectodermal ridge, T, tail, C, cochlea; ED, endolymphatic duct; ES, endolymphatic sac; SC, semicircular canals; V, vestibule. In E10.5 and E11.5 *Six1-/-* inner ears, asterisks identify aberrant dorsal area. In E13.5 *Six1-/-* inner ears, ES* and V* identify endolymphatic sac-like and vestibule-like structures, respectively. a, b and c in panels B-D indicate sections along the latero-medial axis of the same embryo. (E) Representative images of SA-BetaGal staining of E10.5 otic vesicles of the indicated genotypes after 24h in ex-vivo culture conditions. (F) QPCR analysis of the expression of the indicated genes in E11.5 otic vesicles of the indicated genotypes. (G) Genotyping by PCR of *Six1*-cKO mice. Six1 WT, wild-type allele. Six1 LoxP, floxed allele. Six1 del, deleted allele after Cre recombinase action. Cre, Cre transgene.

Figure S2

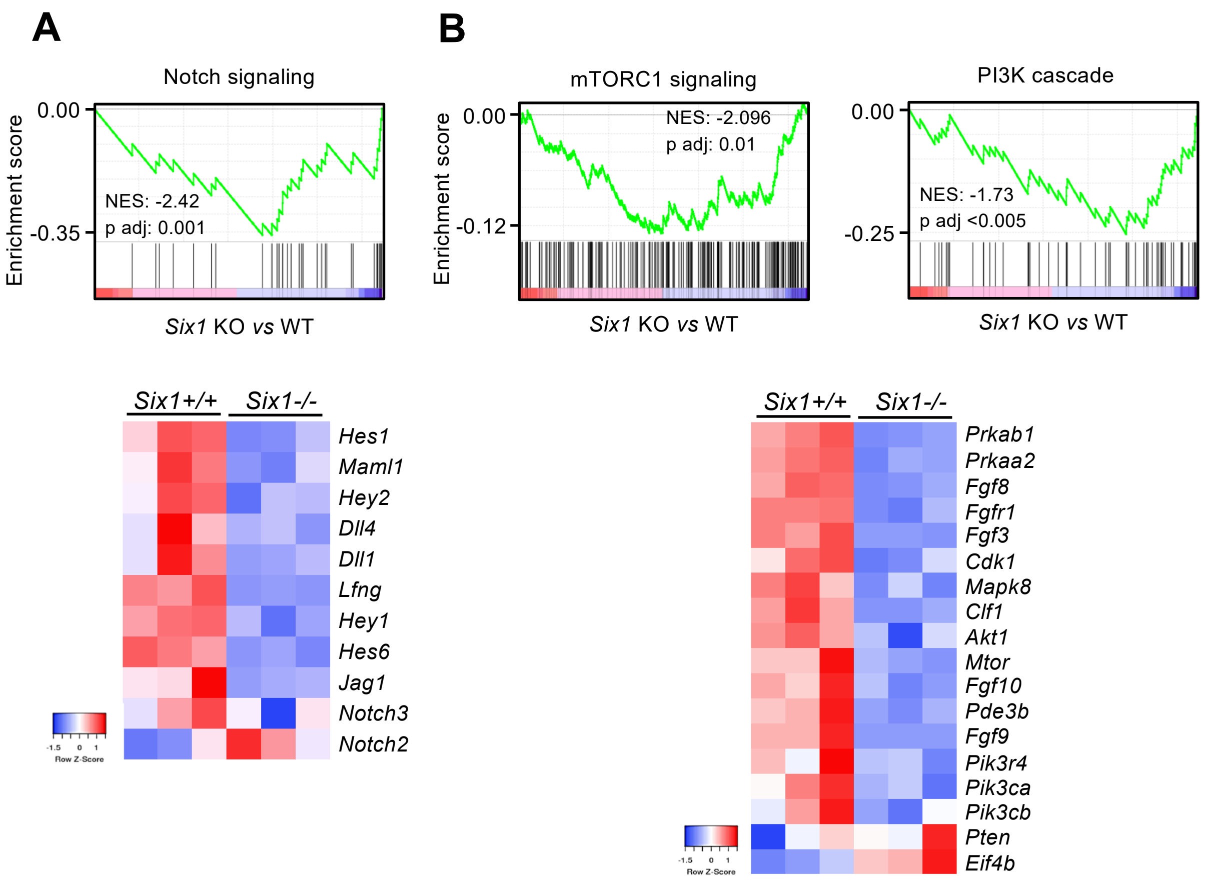

Fig. S2. Notch and PI3K pathways alterations. (A) Enrichment plot (top) for the geneset "Notch Signaling" (Hallmark) and heatmap analysis (bottom) of the expression of Notch pathway genes. (B) Enrichment plot (top) for the genesets "mTORC1 Signaling" (Hallmark) and "PI3K Cascade" (Reactome) and heatmap analysis (bottom) of the expression of PI3K genes. NES, normalized enrichment score.

Development • Supplementary information

Figure S3

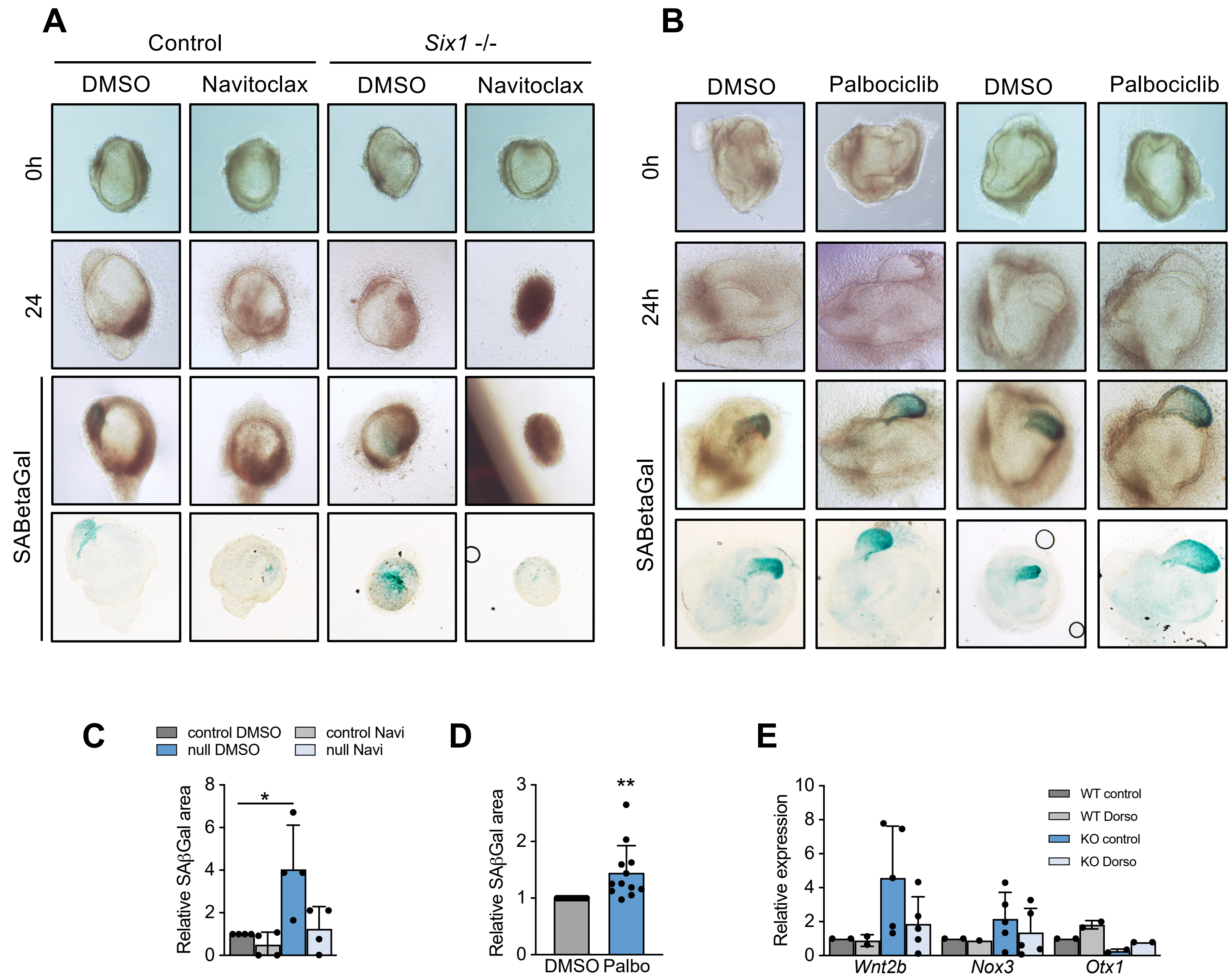

Fig. S3. *Ex vivo* otic vesicle assays. (A) Additional representative images of E10.5 control or *Six1*-null otic vesicles treated with 2 µM Navitoclax or DMSO at 0 and 24 hours and after SA-BetaGal staining in the well (top) or in slides (bottom). (B) Additional representative images of E10.5 wild-type otic vesicles treated with 2 µM Palbociclib or DMSO, as described in A. (C) Quantification of SA-BetaGal-positive area after 24h treatment with Navitoclax (n=4). (D) Quantification of SA-BetaGal-positive area after 24h treatment with Palbociclib (n=10). (E) QPCR analysis of expression of the indicated genes in control and Six1-deficient otic vesicles after 24h treatment with 2 µM Dorsomorphin or DMSO (n=4).

Development • Supplementary information

Table S1. Differential expression data and functional enrichment analysis from the RNA-Seq study of *Six1*-null and wild-type otic vesicles.

Click here to download Table S1

Table S2. Primary antibodies used in this study.

Protein	*Reference*	*Source*	*Dilution*
Ki67	D3B5	Cell Signalling Technology	1:50
p15Ink4b	PAT56B/A7	CNIO Histopathology Core Unit	Undiluted
p21Cip1	291H/B5	CNIO Histopathology Core Unit	Undiluted
Phospho-Histone H3	06-570	Millipore	1:500
SIX1	HPA001893	Sigma	1:500

Table S3. PCR primers used in this study.

Transcript	*Forward*	*Reverse*
Bmp2	CGGACTGCGGTCTCCTAA	GGGGAAGCAGCAACACTAGA
Bmp5	TGACGGGGAATGTTCTTTTCCA	ACATCAGGTGTACCAGGGTCT
Bmp7	AGCTTCGTCAACCTAGTGGAA	TCAAACCGGAACTCCCGATG
Bmp8a	CTGGTCATGAGCTTCGTCAA	AGCAGGGATCTGGGTTAGGT
Cdkn1a (p21)	TCCACAGCGATATCCAGACA	GGACATCACCAGGATTGGAC
Cdkn1b (p27)	GAGCAGTGTCCAGGGATGAG	TCTGTTCTGTTGGCCCTTTT
Cdkn2a (p16)	CGTACCCCGATTCAGGTG	ACCAGCGTGTCCAGGAAG
Cdkn2a (p19Arf)	GGGTTTTCTTGGTGAAGTTCG	TTGCCCATCATCATCACCT
Cdkn2b (p15)	GGCGCCCAATCCAGGTCAT	GTTGGGTTCTGCTCCGTGG
Dlx5	GCTCAATCAATTCCCACCTGC	AGCCCATCTAATAAAGCGTCCC
Gbx2	GCAAGGGAAAGACGAGTCAAA	GGCAAATTGTCATCTGAGCTGTA
Igf1	AAAGTGGTCCTGGCGTGGGTAGATT	TCTACAACATCCATGCATTTTCGGC
Neurod1	AGGAACACGAGGCAGACAAGA	CTCCCCCGTTTCTCAGAGAGT

Development • Supplementary information

Nox3	CGACGAATTCAAGCAGATTGC	AAGAGTCTTTGACATGGCTTTG G
Otx1	GAACCTTCCTTCTCCGAAATCT	GATCTTCACATCGGACAAATCA
Otx2	AATCAACTTGCCAGAATCCAG GG	GCTGTTGGCGGCACTTAGC
Tgfb2	CGAGACCAAATACTTTGCCAC AAAC	CCATGAAGCTTCGGCAGACA
Wnt2b	TAGACACGTCCTGGTGGTACA T	ACCAGACCGGGGATGTTGTC
18s rRNA	CCAGTAAGTGCGGGTCATAAG C	CCTCACTAAACCATCCAATCGG

Development • Supplementary information
