# Supplementary material for: Dysfunction of programmed embryo senescence is linked to genetic developmental defects
Source: Development. 2023 May 3;150(9):dev200903. doi: 10.1242/dev.200903 (PMC10259514; doi:10.1242/dev.200903)
Supplement: Supplementary file 1 [file develop-150-200903_review_history.pdf]

Dysfunction of programmed embryo senescence is linked to genetic developmental defects

Cristina De Lope, Rebeca García-Lucena, Marta Magariños, Yolanda León, Nuria Casa-Rodríguez, Nuria Contreras, Carmen Escudero-Iriarte, Isabel Varela-Nieto, Pascal Maire and Ignacio Palmero
DOI: 10.1242/dev.200903

Editor: Patrick Tam

Review timeline

Original submission:	6 May 2022
Editorial decision:	17 June 2022
First revision received:	15 December 2022
Editorial decision:	31 January 2023
Second revision received:	14 March 2023
Accepted:	20 March 2023

Original submission

First decision letter

MS ID#: DEVELOP/2022/200903

MS TITLE: Dysfunction of programmed embryo senescence is linked to genetic developmental defects.

AUTHORS: Cristina De Lope, Carmen Escudero-Iriarte, Marta Magarinos, Isabel Varela-Nieto, Pascal Maire, and Ignacio Palmero

I have now received all the referees' reports on the above manuscript, and have reached a decision. The referees' comments are appended below, or you can access them online: please go to BenchPress and click on the 'Manuscripts with Decisions' queue in the Author Area.

As you will see, the referees express considerable interest in your work, but have some significant criticisms and recommend a revision of your manuscript before we can consider publication. If you are able to revise the manuscript along the lines suggested, which may involve further experiments, I will be happy receive a revised version of the manuscript. Your revised paper will be re-reviewed by one or more of the original referees, and acceptance of your manuscript will depend on your addressing satisfactorily the reviewers' major concerns. Please note that Development will normally permit only one round of major revision.

If it would be helpful, you are welcome to contact us to discuss your revision in greater detail. Please send us a point-by-point response indicating your plans for addressing the referee's comments, and we will look over this and provide further guidance.

Please attend to all of the reviewers' comments and ensure that you clearly highlight all changes made in the revised manuscript. Please avoid using 'Tracked changes' in Word files as these are lost in PDF conversion. I should be grateful if you would also provide a point-by-point response detailing how you have dealt with the points raised by the reviewers in the 'Response to Reviewers' box. If you do not agree with any of their criticisms or suggestions please explain clearly why this is so.

© 2023. Published by The Company of Biologists under the terms of the Creative Commons Attribution License (https://creativecommons.org/licenses/by/4.0/).

Reviewer 1

Advance summary and potential significance to field

Palmero and col used Six1-deficient mice, an animal model of the Branchio-Oto-Renal (BOR) syndrome, to show aberrant senescence and defective morphogenesis in the inner ear. Also they linked this aberrant senescence to the TGF-beta/BMP pathway. These results show that altered developmental senescence may be linked to developmental disorders.

Comments for the author

Issues that must first be addressed:
1- In Six1-null otic vesicles ED is absent but with palbociclib it looks enlarged, Explain why palbociclib does not reproduce the Six1 phenotype in this case. This contradicts lane 231-2: "Treatment with Palbociclib led to morphological changes reminiscent of the Six1-deficient phenotype"
2- The authors suggest that Six1 may act as a physiological repressor of senescence during inner ear development. However, the trigger of the senescence phenotype remains unknown. This anti-senescence effect could have been tested by treating Six1 deficient otic vesicles with palbociclib, which should result in higher senescence burden if this model is right.
3- Why palbociclib and not Bmp8a was chosen to induce senescence?

Minor comments
4- Lane 112: this is not clear "without an elongated forming endolymphatic duct'
5- Lane 120 and Figure 1A: Please use arrows to identify the neural tube, the apical ectodermal ridge and the tip of the tail
6- Figure 1: Please define D, P and M
7- Figure 4F, identify the endolymphatic sac, only ED is identified.

Reviewer 2

Advance summary and potential significance to field

This study aims to further our understanding of the role that embryonic cellular senescence may contribute to normal and aberrant embryonic development. This is an important and understudied area of developmental biology that warrants further investigation and may provide novel insights into the aetiology of certain congenital abnormalities.

The authors approach this using mutant mice deficient for Six1 (a previously described negative regulator of adult cellular senescence), which results in a developmental phenotype recapitulating the defects found in human Branchio-Oto-Renal (BOR) syndrome. The prior discovery of cellular senescence occurring embryonically in serval tissues, including the developing renal and auditory systems, which are malformed in BOR syndrome, creates a compelling basis to investigate if cellular senescence is involved in this syndrome.
The authors provide evidence that loss of Six1 disrupts the normal levels of embryonic cellular senescence in the developing inner ear, resulting in morphological defects that resemble a BOR syndrome phenotype. In my view, these results are interesting, novel and provide insight into the cellular and molecular mechanisms underlying BOR syndrome defects. Therefore, I would endorse this article for publication in Development following minor revision.

Comments for the author

The authors provide evidence that absence of Six1 expression expands the domain of embryonic senescence found in otic vesicles during development. This was assayed through quantification of SA-Beta-Gal and PH3 stained areas at serval developmental timepoints during inner ear morphogenesis. Could the authors add a section in the materials and methods elaborating on the quantification of the stains used. Specifically how the area was chosen and delineated and how many sections were analysed per embryos.

© 2023. Published by The Company of Biologists under the terms of the Creative Commons Attribution License (https://creativecommons.org/licenses/by/4.0/).

For the transcriptomic experiments (detailed in Figure 2) it appears unclear to me why the authors decided to carry out the experiment at 10.5 dpc, rather than a later developmental stage when the markers of cellular senescence and the morphological phenotypes are more pronounced? Could the authors explain why this stage was chosen? Later timepoints may have shown a more pronounced senescence signature. It would be good to include further qPCR analysis of the studied senescence markers at later developmental timepoints in wild type and Six1-deficient otic vesicles. Following from the observed expansion of SA-B-Gal reactivity at later stages, it would be assumed that the expression of the senescence markers would increase.

For the navitoclax experiments (Figure 4), it would be good to include staining for apoptotic markers such as cleaved caspase-3, which would be expected to be higher in abundance in SA-Beta-Gal positive areas. Quantification of the area of SA-Beta-Gal positivity should be added for these experiments in Figure 4B and F.

The Text states that, "Navitoclax-treated Six1-deficient vesicles showed similar changes in shape and size and the distinctive enlarged SA-BetaGal positive dorsal region was no longer distinguishable, supporting its link to dysregulated senescence (Figure 4B,C and S3A)", however Six1-deficient navitoclax treated otic vesicles appear to have similar SA-Beta-Gal positivity and morphology to DMSO treated (Figure 4B). Could the authors please check these images. Similarly, the Palbociclib treated otic vesicles in Figure 4F show unconvincing differences in SA-Beta-Gal positivity. Quantification should be added for this experiment also.
Downregulation of p21 (by qPCR or immunohistochemical analysis) would be a good internal control for the navitoclax treatment experiment in Figure 4B.
Quantification of proliferation by PH3 staining would be a good addition to the results presented in Figure 4, which would be expected to be increased in navitoclax treated and decreased in Palbociclib treated.

Minor comment - Missing word "ear on line 215 after the word inner.

<u>Reviewer 3</u>

Advance summary and potential significance to field

The authors investigate Six1 deficient mice, as a model of Branchio-Oto-Renal
(BOR) syndrome, and describe how these mice have increased cellular senescence in the developing ear making a link with senescence and developmental defects. Senescence is usually associated with aging and disease, so the finding that it could be involved in developmental defects seems very interesting, and represents a significant advance.

Comments for the author

Overall the work appears to be of high quality, but additional experiments are needed to substantiate some of the claims.

Regarding the use of Six1-deficient mice as a model for BOR syndrome, the study would benefit from further references about how this is a model for the disease. Or is this more of an association? If this is indeed a model of the syndrome, did the authors see any alterations in senescence in the other affected tissues? It is understood that the focus is on the developing ear, but further association, if possible, would strengthen the claims.

More details should be provided for the quantification of the SABgal area. And for PHH3, it is probably better to represent the percentage of cells, rather than area.

The authors should describe in the text which samples were used and how they were prepared for the RNAseq. It's a pity the authors chose E10.5 to perform the RNAseq rather than E11.5 where the SA-BGAL is stronger and the phenotype more obvious?

The induction of the senescence genes seems quite low. As p16/p19 are often regarded among the most reliable markers of senescence, this should be discussed/explained. How does their expression appear at later stages (E11.5, E13.5)?

© 2023. Published by The Company of Biologists under the terms of the Creative Commons Attribution License (https://creativecommons.org/licenses/by/4.0/).

Also, the replicates for QPCR validation seem low. Even though pooled samples are used, n=2 replicates is too few. What statistical tests are performed on this?

If possible, this result would be strengthened with staining or in situ hybridization. Although unfortunately it is known there is no good antibody for mouse p16. Were any other markers of senescence examined? (gH2AX LaminB…..)

The increased expression of p21 is not fully clear from the images, and the wildtype seems to show more co-expression with SABgal than the Six1-deficient mice. Also, its curious that there does not appear to be any p21 expression in the mutant at E11.5 (Supp Fig 1B), although by RNAseq it is increased at E10.5. Perhaps western blotting for p21 at different timepoints could help confirm the increase?

Did the authors consider cross the Six1-deficient with p21-deficient mice. This would be a very strong supporting result if there were differences/rescue. In addition or alternatively, the use of BMP inhibitors to perturb senescence or its markers would strongly support the claims.

For the ex vivo experiments, they should quantify the SABgal with/without with Navitoclax. It appears that the effect is stronger on control embryos than it is on Six1-deficient. Also, as navitoclax is a suggested senolytic did the authors see any increase in cell death in the senescent cells? And how did the senescence markers change in response to drug. The ex vivo culture and senolytic experiments would benefit from additional characterization.

The text says the control embryos for the Navitoclax experiment consisted of wildtype and heterozygous. Can you be sure there is no dose effect on senescence induction in the Six1 hets?

For most of the quantifications throughout the manuscript, the individual samples should be included and visible, rather than just a plain bar-graph.

A discussion point: the authors state that Six1 deficiency alters the physiological program of developmental senescence (and in the title). However, in their model, additional senescence genes are expressed. Is this really an alteration of developmental senescence, or an induction of additional ectopic senescence?

First revision

<u>Author response to reviewers' comments</u>

Point-by-point response to reviewers' comments Reviewer 1

We are grateful to the reviewer for his/her comments.

Response to specific points

1. In Six1-null otic vesicles ED is absent but with palbociclib it looks enlarged, Explain why palbociclib does not reproduce the Six1 phenotype in this case. This contradicts lane 231-2: “Treatment with Palbociclib led to morphological changes reminiscent of the Six1-deficient phenotype”

We apologize if we failed to convey clearly our interpretation of these results. Six1-null otic vesicles lack a distinct senescent endolymphatic duct (ED) in their dorsal end. Instead, a large portion of the dorsal region of the vesicles acquires features of the ED, as shown by increased senescence markers (IHC and QPCR) or augmented expression of the ED marker Wnt2b (QPCR). We agree that Palbociclib treatment of ex vivo vesicles may not reproduce exactly the Six1-null phenotype, but we consider that the increase in ED-like area and the gene expression changes caused by Palbociclib in wild type vesicles (Figure 4F-H) are highly reminiscent to those in Six1-null vesicles. We believe that these results support the involvement of senescence in the Six1 phenotype. It should be borne in mind that Palbociclib is used here as an acute inducer of

© 2023. Published by The Company of Biologists under the terms of the Creative Commons Attribution License (https://creativecommons.org/licenses/by/4.0/).

senescence in vesicles where ED formation is already under way (in our hands vesicles of earlier stages are not viable for ex-vivo assays). This situation is radically different from Six1-null embryos, where the senescence program may be disrupted throughout the process of inner ear morphogenesis. Also, Palbociclib is used on these assays as a general senogenic agent, in order to support a link to senescence, but it is possible that Palbociclib does not fully reproduce the molecular changes associated to Six1 deficiency.

2. The authors suggest that Six1 may act as a physiological repressor of senescence during inner ear development. However, the trigger of the senescence phenotype remains unknown. This anti-senescence effect could have been tested by treating Six1 deficient otic vesicles with palbociclib, which should result in higher senescence burden if this model is right.

Since our hypothesis is that Six1 deficiency leads to augmented senescence, we reasoned it would be more informative to test if a senolytic treatment (Navitoclax) could revert the phenotype of Six1-deficient otic vesicles and, conversely, if a senogenic treatment (Palbociclib) could induce in wild-type vesicles a phenotype similar to Six1- deficient otic vesicles. Collectively, we believe that the data shown in Figure 4 supports the notion that the gene expression and morphological changes found in Six1-deficient otic vesicles are linked to senescence. Unfortunately, the limited availability of otic vesicles of all genotypes has not made possible to perform the experiment suggested by the reviewer.

3. Why palbociclib and not Bmp8a was chosen to induce senescence?

Palbociclib was chosen in these experiments as a general senogenic agent, with the purpose to test the link between senescence and morphogenic defects in this setting (See also response to point 1). To address the potential role of Bmp8a in this phenotype, we have performed experiments to study the effects of recombinant BMP8A in ex vivo otic vesicles. Unfortunately, these experiments have failed to yield a clear answer. Using several concentrations of commercial recombinant human BMP8A protein (#1073-BPC, R&D systems) we obtained a limited biological response with high variability. Only a small fraction of BMP8A-treated vesicles (22%, 5/22) showed increased levels of Id1, a general marker of BMP activity, and this increase was generally marginal. Using Id1 as a surrogate marker of BMP8A activity, we analyzed several relevant markers by QPCR in the subset of vesicles with increased Id1. As shown in Figure 1 of this rebuttal, several genes elevated in Six1-null inner ear (linked to morphogenesis or senescence) showed a tendency to increase upon BMP8A treatment but these differences never reached statistical significance due to the high variability. Similarly, analysis of the morphology also revealed high variability, with a small fraction of BMP8A-treated vesicles showing increased ED area (Figure 1 of the rebuttal). Our results are consistent with previous reports highlighting the limited bioactivity of commercially available BMP8A (Wu, Reproduction, 2020). Despite these limitations, these experiments appear to suggest that exogenous BMP8A might mimic at least some of the morphological and gene expression features of Six1-null inner ears. However, due to their poor reproducibility, we believe that at this stage the data is not suitable to be part of the manuscript and is only included in this rebuttal for inspection by the reviewers. Further experiments with alternative sources of BMP8A would be necessary to firmly establish the direct role of BMP8A in this context. Due to these technical difficulties, we have used an alternative approach based on the use of the BMP inhibitor drug Dorsomorphin (see response to point 10 of reviewer 3).

Minor comments:

4. Lane 112: this is not clear “without an elongated forming endolymphatic duct’

We apologize for the wording used. The sentence has been replaced by “(…) without a distinct elongated endolymphatic duct” to improve clarity (line 117 in the revised version).

5. Lane 120 and Figure 1A: Please use arrows to identify the neural tube, the apical ectodermal ridge and the tip of the tail.

We thank the referee for this suggestion. We believe that including all the required information in the same panel might be confusing. For this reason, we have included in the Supplementary

© 2023. Published by The Company of Biologists under the terms of the Creative Commons Attribution License (https://creativecommons.org/licenses/by/4.0/).

Figures section larger magnification images of whole mount SA-BetaGal staining of embryos at the same stages, showing the indicated senescent structures.

Reviewer 2

We are grateful to the reviewer for his/her comments.

Response to specific points

1. The authors provide evidence that absence of Six1 expression expands the domain of embryonic senescence found in otic vesicles during development. This was assayed through quantification of SA-Beta-Gal and PH3 stained areas at serval developmental timepoints during inner ear morphogenesis. Could the authors add a section in the materials and methods elaborating on the quantification of the stains used. Specifically how the area was chosen and delineated and how many sections were analysed per embryos.

We apologize if this information was not sufficiently clear in the previous version. The method used for quantification, including how the areas were delineated and the number of sections used per embryo is now explained in more detail in Supplemental Material and Methods section in the paragraphs "Senescence-Associated Beta-Galactosidase staining" and "Immunohistochemistry in paraffin".

2. For the transcriptomic experiments (detailed in Figure 2) it appears unclear to me why the authors decided to carry out the experiment at 10.5 dpc, rather than a later developmental stage when the markers of cellular senescence and the morphological phenotypes are more pronounced? Could the authors explain why this stage was chosen? Later timepoints may have shown a more pronounced senescence signature.

The decision to perform the transcriptomic experiments with E10.5 embryos was based on technical reasons. At E10.5, otic vesicles of both genotypes are compact structures without an elongated endolymphatic duct (ED) and they can be easily dissected from the embryos without surrounding mesenchyme. Dissection of wild-type vesicles of later stages with intact ED and no mesenchyme is technically challenging and we were concerned that the marked morphological differences between genotypes at these stages (see Figure 1B of the manuscript) might result in different levels of mesenchymal contamination that could obscure the transcriptomic results. We fully agree that later stages may have been more informative, due to the more pronounced expression of senescence markers. However, we consider that the fact that we find a significant enrichment of a senescence-related gene signature in Six1-deficient E10.5 embryos supports the notion that the senescence program is disrupted since very early stages in mutant embryos.

3. It would be good to include further qPCR analysis of the studied senescence markers at later developmental timepoints in wild type and Six1-deficient otic vesicles. Following from the observed expansion of SA-B-Gal reactivity at later stages, it would be assumed that the expression of the senescence markers would increase.

To address this question, we have performed QPCR analysis of wild-type, Six1-/- and Six1+/- otic vesicles freshly isolated from E11.5 embryos (Supplementary Figure S1F). We observed the differential expression of several key deregulated genes in E11.5 Six1-/- vesicles relative to wild-type littermates. In addition, the QPCR data of untreated vesicles from ex vivo experiments 24h after extraction from E10.5 embryos (which may be considered equivalent to E11.5 embryos) also show similar gene expression profiles to freshly isolated E10.5 vesicles (compare wild-type/control and KO/control samples in Figure 4D and Figure S3). Extraction of intact E13.5 inner ears, where we would expect increased expression of senescence markers, is technically very challenging and has not been attempted.

4. For the navitoclax experiments (Figure 4), it would be good to include staining for apoptotic markers such as cleaved caspase-3, which would be expected to be higher in abundance in SA-Beta-Gal positive areas.

© 2023. Published by The Company of Biologists under the terms of the Creative Commons Attribution License (https://creativecommons.org/licenses/by/4.0/).

Unfortunately, due to the limited number of vesicles obtained for each genotype, it has not been possible to perform the requested assay.

5. Quantification of the area of SA-Beta-Gal positivity should be added for these experiments in Figure 4B and F.

We have quantified the SA-BetaGal-positive areas in Figure 4B and F as requested. These graphs are included in Supplementary Figure S3C,D.

6. The Text states that, "Navitoclax-treated Six1-deficient vesicles showed similar changes in shape and size and the distinctive enlarged SA-BetaGal positive dorsal region was no longer distinguishable, supporting its link to dysregulated senescence (Figure 4B,C and S3A)", however Six1-deficient navitoclax treated otic vesicles appear to have similar SA-Beta-Gal positivity and morphology to DMSO treated (Figure 4B). Could the authors please check these images.

We have included a new version of this figure with new panels of SA-BetaGal stainings, which we believe are more representative of the phenotype observed. Together with the new quantification of SA-BetaGal-positive area now included in Supplementary Figure S3C, and additional examples shown in in Supplementary Figure S3A we believe that the data in the revised version clearly supports the conclusion that treatment with the senolytic Navitoclax provokes significant changes in morphology and SA-BetaGal staining in vesicles of both genotypes.

7. Similarly, the Palbociclib treated otic vesicles in Figure 4F show unconvincing differences in SA-Beta-Gal positivity. Quantification should be added for this experiment also.

We have quantified the SA-BetaGal-positive areas in Figure 4B and F as requested. These graphs are included in Supplementary Figure S3C,D. Additional examples of Palbociclib treated vesicles are also shown in Supplementary Figure S3B.

8. Downregulation of p21 (by qPCR or immunohistochemical analysis) would be a good internal control for the navitoclax treatment experiment in Figure 4B.

We have performed the requested analysis but we failed to observe a significant change in p21 after Navitoclax treatment. In line with our observations, similar experiments with Navitoclax in chicken otic vesicles (Gibaja, Sci Rep, 2019) also failed to show significant changes in p21 transcript levels by QPCR. One possible explanation for our result could be related to the complex pattern of p21 expression in inner ear at the stages studied. In addition to the senescence-associated positive cells, p21-positive cells can also be detected by IHC in non-senescent prosensory regions at E11.5 and E13.5 (Figure 2D and S1D). While these two populations of p21-positive cells can be distinguished in IHC, this is not possible in QPCR analysis of whole vesicles. It is likely that a similar p21 expression pattern might be present in vesicles at the end of the ex-vivo culture experiments (equivalent to E11.5 stage). For this reason, potential changes in p21 expression linked to senescence might be obscured by the presence of non-senescent p21-expressing cells.

9. Quantification of proliferation by PH3 staining would be a good addition to the results presented in Figure 4, which would be expected to be increased in navitoclax treated and decreased in Palbociclib treated.

Unfortunately, due to the limited availability of vesicles of all genotypes, it has not been possible to perform the requested assay.

Reviewer 3

We are grateful to the reviewer for his/her comments.

Response to specific points

© 2023. Published by The Company of Biologists under the terms of the Creative Commons Attribution License (https://creativecommons.org/licenses/by/4.0/).

1. Regarding the use of Six1-deficient mice as a model for BOR syndrome, the study would benefit from further references about how this is a model for the disease. Or is this more of an association?

The evidence that supports that Six1-deficient mice can be considered an animal model for BOR syndrome is as follows. 1) Genetic analysis has shown that mutations in genes of the functional SIX/EYA pathway are the most frequent genetic alterations in BOR patients (EYA1, approx. 40% of cases; SIX1 and SIX5, approx. 5% each). 2) The Six1- null strain used in this study (Laclef,. Development, 2003) and other Six1-deficient models (Bosman, Dev Biol, 2009) display phenotypes that recapitulate the major BOR syndrome symptoms, most notably defects in the auditory system (outer, middle and inner ear; Bosman, Dev Biol, 2009, Laclef, Mech Dev, 2003, Ozaki, Development, 2004, Zheng, Development, 2003) and the renal system (mesonephros and metanephros, Xu, Development, 2003, Kobayashi, Mech Dev, 2007). Consistent with the notion that disruption of the functional SIX/EYA pathway is causative for BOR syndrome, very similar ear and kidney phenotypes have been described in mice deficient for other genes of the pathway, most notably Eya1 (Xu, Nat Gen, 1999). Collectively, we believe that these facts support the notion that Six1-deficient mice can be considered mouse models for the BOR syndrome. Due to length limitations, we have kept unchanged the relevant paragraph and, as suggested, we have included in the manuscript an additional reference to support our statement (Bosman, Dev Biol, 2009).

2. If this is indeed a model of the syndrome, did the authors see any alterations in senescence in the other affected tissues? It is understood that the focus is on the developing ear, but further association, if possible, would strengthen the claims.

We thank the referee for this suggestion. To answer this question, we have recently initiated the study of the mesonephros, since this embryonic kidney shows physiological senescence (Muñoz-Espín, Cell, 2013) and is also altered in Six1-deficient embryos (Kobayashi, Mech Dev, 2007). At this point we have only been able to obtain preliminary data about senescence in this organ in Six1-null embryos, as shown in Figure 2 of this rebuttal. The analysis of a limited number of E11.5 embryos suggests a tendency to increased SA-BetaGal activity and reduced proliferation (measured with phospho- histone H3) in mesonephric tubules of Six1-deficient embryos, relative to age-matched wild-type embryos. No clear differences were observed for p21 positivity. We believe that these preliminary results are promising and we intend to pursue this study further. However, at this point we consider that the results are too preliminary to form part of the manuscript and we have included them in this rebuttal only for reviewers' inspection.

3. More details should be provided for the quantification of the SABgal area. And for PHH3, it is probably better to represent the percentage of cells, rather than area.

Details for the quantification of the SA-BetaGal area have been included in the new version of Supplemental Material and Methods.

4. The authors should describe in the text which samples were used and how they were prepared for the RNAseq. It's a pity the authors chose E10.5 to perform the RNAseq rather than E11.5 where the SA-BGAL is stronger and the phenotype more obvious?

The protocol used for RNASeq, including the samples used and how they were prepared is described in the Supplemental Materials and Methods section. See also the response to point 2 of reviewer 2 regarding the embryo stage used for the RNASeq experiments.

5. The induction of the senescence genes seems quite low. As p16/p19 are often regarded among the most reliable markers of senescence, this should be discussed/explained. How does their expression appear at later stages (E11.5, E13.5)?

To address this question, we performed IHC for p16Ink4a with a validated antibody. However, this protein was undetectable at all the stages analyzed in inner ears of both genotypes (data not shown), in line with published data for wild-type embryos (Muñoz- Espin, Cell, 2013). As a complementary genetic strategy to address the potential involvement in this phenotype of p16 and p19Arf (proteins encoded in the Cdkn2a locus), we have generated a double mutant colony

© 2023. Published by The Company of Biologists under the terms of the Creative Commons Attribution License (https://creativecommons.org/licenses/by/4.0/).

crossing Six1-heterozygous mice with Cdkn2a-heterozygous mice (Serrano, Cell, 1996). At this stage, we have been able to analyze a limited number of embryos. Assessment of inner ear morphology and proliferation rate (Ki67) did nor reveal any clear difference between Six1-/-;Cdkn2a+/+ and Six1-/-;Cdkn2a-/- embryos (Figure 3A of this rebuttal). These preliminary results do not support a critical role for the Cdkn2a locus in the Six1-associated phenotypes analyzed (see also response to point 9). We believe that these results are too preliminary to reach conclusions and we would rather not include them in the manuscript.

6. Also, the replicates for QPCR validation seem low. Even though pooled samples are used, n=2 replicates is too few. What statistical tests are performed on this?

Each replicate used for QPCR validation, represents a pool of RNA from six vesicles obtained from three independent embryos. The statistical tests used are described in the Supplemental Material and Methods section. Unfortunately, the limited availability of otic vesicles of both genotypes for the different assays have not allowed us to increase the sample size in this analysis. In any case, we believe that the good general agreement between QPCR data and RNASeq results and the comparable expression pattern for a selection of genes in ex vivo analysis supports the validity of these QPCR results.

7. If possible, this result would be strengthened with staining or in situ hybridization. Although unfortunately it is known there is no good antibody for mouse p16. Were any other markers of senescence examined? (gH2AX, LaminB…..)

We have attempted in situ hybridization but, unfortunately, this approach has been unsuccessful so far. As mentioned in the response to point 5, p16 IHC failed to detect specific signal at any stage, in agreement with published data (Muñoz-Espín, Cell, 2013). We have also tried other markers previously associated to developmental senescence but, so far, we have not been able to observe consistent association to senescence. To further support the link to senescence, we have now added to the heatmap panel in Figure 2 of the new version the expression data of genes associated to lysosomal activity, a well-established hallmark of senescent cells.

8. The increased expression of p21 is not fully clear from the images, and the wildtype seems to show more co-expression with SABgal than the Six1-deficient mice. Also, its curious that there does not appear to be any p21 expression in the mutant at E11.5 (Supp Fig 1B), although by RNAseq it is increased at E10.5. Perhaps western blotting for p21 at different timepoints could help confirm the increase?

In the images shown in Figure 2D, the SA-BetaGal staining is somewhat more intense in the wild-type embryos. This may give the impression that the level of co-expression p21/SA-BetaGal could differ between genotypes. However, the analysis of several independent embryos of both genotypes indicates that this is not the case. Accordingly, we are confident that the increased area of senescence-associated p21 shown in the quantification panel reflects increased senescent areas in Six1-null embryos, consistently with other senescent markers. Also, please see the response to point 8 of reviewer 2 regarding p21 analysis by QPCR in our model.

9. Did the authors consider cross the Six1-deficient with p21-deficient mice. This would be a very strong supporting result if there were differences/rescue.

We thank the reviewer for this suggestion. We have generated a double transgenic mouse strain crossing heterozygous Six1 cKO mice with Cdkn1a +/- mice (Brugarolas, JCB, 1998). At this point we have been able to analyze a limited number of E11.5 embryos from this colony where we evaluated inner ear morphology, SA-BetaGal activity and proliferation (Phospho-H3 or Ki-67). These preliminary results have not revealed so far a clear reversion of the parameters analyzed in Six1-/-;Cdkn1a-/- embryos (Figure 3B of this rebuttal). Similarly to the data with Cdkn2a-null mice described above, we are including here representative images for inspection of the reviewers but we believe that these results are too preliminary at this stage to reach statistically significant conclusions and it would be more adequate not to include them in the manuscript.

10. In addition or alternatively, the use of BMP inhibitors to perturb senescence or its markers would strongly support the claims.

© 2023. Published by The Company of Biologists under the terms of the Creative Commons Attribution License (https://creativecommons.org/licenses/by/4.0/).

To address this question, we have performed experiments in ex vivo vesicles using Dorsomorphin, an inhibitor of the SMAD1/5/8 pathway which has been shown to revert effects of Bmp8a in other biological settings (Wu, Science Signaling, 2017; Wu, Reproduction, 2020). In these experiments, we have observed that Dorsomorphin treatment for 24h could revert Six1-associated changes in transcript levels for some of the most differentially expressed genes, although these differences did not reach statistical significance (Supplemental Figure S3E). We believe that these results support the notion that deregulation of BMP signaling (most likely mediated by Bmp8) is, at least in part, responsible for the aberrant phenotypes observed in Six1-deficient inner ear. A mention to these new results is included in the revised version of the manuscript.

11. For the ex vivo experiments, they should quantify the SABgal with/without with Navitoclax. It appears that the effect is stronger on control embryos than it is on Six1- deficient. Also, as navitoclax is a suggested senolytic, did the authors see any increase in cell death in the senescent cells? And how did the senescence markers change in response to drug. The ex vivo culture and senolytic experiments would benefit from additional characterization.

Please see responses above to related comments (points 4 to 9) of reviewer 2.

12. The text says the control embryos for the Navitoclax experiment consisted of wildtype and heterozygous. Can you be sure there is no dose effect on senescence induction in the Six1 hets?

To address this question, the current version includes additional evidence supporting the similarity between wildtype and heterozygous embryos. Supplemental Figure S1C includes immunohistochemical data, present in in the previous version, that shows that there are no overt differences in morphology or Six1 expression between wildtype and heterozygous inner ears at E13.5, the stage where the morphological impact of Six1 deletion is most obvious. We have now reinforced this observation including new items: images of E10.5 otic vesicles after 24h ex vivo culture, highlighting similarities in morphology and SA-BetaGal staining (Figure S1E) and QPCR data of a selection of representative genes in E11.5 otic vesicles (Figure S1F). Collectively, these results support the conclusion that there is no distinguishable dose effect in the morphological and senescence markers analyzed between wildtype and heterozygous embryos.

13. For most of the quantifications throughout the manuscript, the individual samples should be included and visible, rather than just a plain bar-graph

We have changed the layout of all the graphs to make visible the data from individual samples, according to the reviewer's request.

14. A discussion point: the authors state that Six1 deficiency alters the physiological program of developmental senescence (and in the title). However, in their model, additional senescence genes are expressed. Is this really an alteration of developmental senescence, or an induction of additional ectopic senescence?

The reviewer raises an interesting issue. We believe that the most likely explanation for the phenotype observed is that Six1 participates in the physiological regulation of senescence in the developing inner ear, contributing to define the specific senescent domain within the organ. Accordingly, Six1 deficiency alters the normal program of developmental senescence. However, we cannot formally rule that induction of ectopic senescence also plays a role. We have added a sentence to the Discussion section to acknowledge this question.

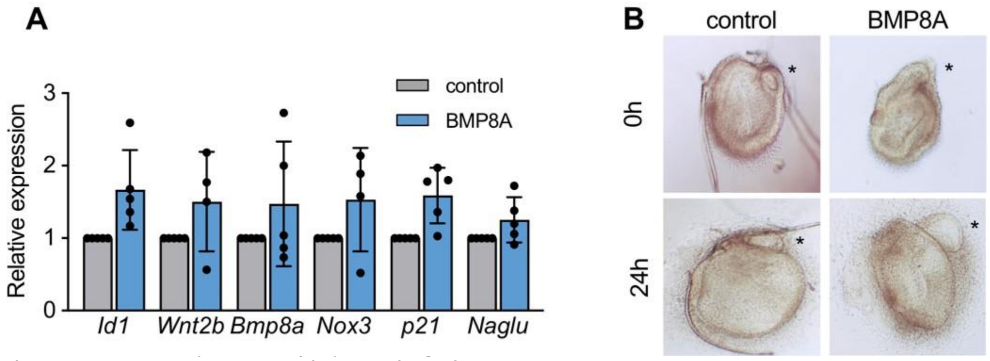

License
(https://creativecommons.org/licenses/by/4.0/).

Figure 1: A. QPCR analysis of the indicated genes in E10.5 wid-type otic vesicles after 24 hours of ex vivo treatment with 500 ng/ml BMP8A or untreated (control). B. Morphology of vesicles treated as in panel A.

NOTE: We have removed unpublished data that had been provided for the referees in confidence.

Second decision letter

MS ID#: DEVELOP/2022/200903

MS TITLE: Dysfunction of programmed embryo senescence is linked to genetic developmental defects.

AUTHORS: Cristina De Lope, Rebeca Garcia-Lucena, Marta Magariños, Yolanda Leon, Nuria Casa-Rodriguez, Nuria Contreras, Carmen Escudero-Iriarte, Isabel Varela-Nieto, Pascal Maire, and Ignacio Palmero

I have now received all the referees reports on the above manuscript and have reached a decision. The referees' comments are appended below, or you can access them online: please go to BenchPress and click on the 'Manuscripts with Decisions' queue in the Author Area.

It was noted by the reviewers that the manuscript has been improved through revision and addressing the concerns raised in the previous review. However, the reviewers opined that critical functional understanding on the impact of mis-regulation of senescence mediator/s on driving the Six1 phenotype is lacking. We do recognize that the requisite experiments might not be feasible due to the unavailability of materials and technical capability. In this case, it would be appropriate to acknowledge, as suggested by Reviewer 1, the limitation of the present study and identify the scope of follow-up work that would address the outstanding issues. As the overall evaluation is positive, we would consider publishing a revised manuscript that has incorporated a discussion of the limitation and issues to be addressed.If you do not agree with this suggestion and the critiques raised by Reviewer 3, please explain clearly why this is so.

Reviewer 1

Advance summary and potential significance to field

The authors provided a reasonable explanation to the points I raised in the first round. They did not succeed in inducing senescence using BMP8a but included a new figure with ex vivo vesicles using Dorsomorphin, an inhibitor of Bmp8a that address my point. I did notice that they could not answer several criticisms from the other reviewers due to technical limitations.

Comments for the author

Authors could include a limitation section in the discussion to acknowledge their inability to perform several requested experiments.

Reviewer 2

Advance summary and potential significance to field

This study aims to further our understanding of the role that embryonic cellular senescence may contribute to normal and aberrant embryonic development. This is an important and understudied

© 2023. Published by The Company of Biologists under the terms of the Creative Commons Attribution License (https://creativecommons.org/licenses/by/4.0/).

area of developmental biology that warrants further investigation and may provide novel insights into the aetiology of certain congenital abnormalities.
The authors approach this using mutant mice deficient for Six1 (a previously described negative regulator of adult cellular senescence), which results in a developmental phenotype recapitulating the defects found in human Branchio-Oto-Renal (BOR) syndrome. The prior discovery of cellular senescence occurring embryonically in serval tissues, including the developing renal and auditory systems, which are malformed in BOR syndrome, creates a compelling basis to investigate if cellular senescence is involved in this syndrome.
The authors provide evidence that loss of Six1 disrupts the normal levels of embryonic cellular senescence in the developing inner ear, resulting in morphological defects that resemble a BOR syndrome phenotype. In my view, these results are interesting, novel and provide insight into the cellular and molecular mechanisms underlying BOR syndrome defects. Therefore, I would endorse this article for publication in Development.

Comments for the author

I would like to thank the authors for the submission of their revised manuscript.
The authors were able to address the majority of the questions I posed, which I feel strengthens the quality of the data and the robustness of the conclusions drawn.
While there are some revisions I requested that were not able to be completed due to the availability of the biological material, I do not feel their absence significantly detracts from the overall conclusions of the study. I am happy to recommend this article for publication in Development.

Reviewer 3

Advance summary and potential significance to field

The authors provide their responses to the reviewer’s questions. Unfortunately, in a number of cases, it appears they were unable to collect enough additional samples to support the questions.

Overall, this is a potentially interesting study, and the suggested link between senescence and the developmental phenotypes in the Six1 mutants is exciting. Unfortunately, however, the data supporting that senescence is a key driver of the phenotype is still not fully convincing.

Ideally, a genetic rescue or partial rescue would have provided key support, but its deficiency in the absence of key senescence mediators (eg Cdkn2a, p21) does not support the claim.

Comments for the author

There is a clear phenotype in the absence of six1, which appears to validate previous findings. This is also associated with some changes in SABgal staining, and the RNAseq appears to support mild gene-expression changes in some key senescence genes (the fold change is small, but in many cases, is not validated by QPCR, to a significant degree)

Therefore, there are still gaps in the understanding. There is suggested increase of p21 transcript at E10.5 but no protein shown at this time. P21 staining is shown at 11.5, but not associated with SABgal. p21 does seem to be increased at 13.5, but the association with SABgal is not clear. Unfortunately, there is no genetic rescue hinted at from preliminary data, suggesting that p21 is not a key mediator.

While it does appear there is some effect of the senolytic drug Navitoclax in the ex vivo cultures, that this is specific for an effect on senescence is not clear. No effect on senescence genes was found/shown, and no evidence for increased cell death in senescent cells is provided.

The inverse approach of inducing senescence with Palbociclib, a known senescence mediator, is also interesting. But this more so supports that a drug that induces senescence in the embryo might cause developmental patterning defects, rather than supporting key roles for senescence caused by Six1 deficiency.

© 2023. Published by The Company of Biologists under the terms of the Creative Commons Attribution License (https://creativecommons.org/licenses/by/4.0/).

Overall, this study would need additional functional results to convincingly support that the phenotypes are caused by alterations in senescence.

Second revision

Author response to reviewers' comments

Authors' response to reviewers' comments

Reviewer 1. Comments for the Author:

Authors could include a limitation section in the discussion to acknowledge their inability to perform several requested experiments.

We are grateful to the reviewer for their suggestion. A new sentence has been added to the Discussion acknowledging the limitations of our study (see also below the response to reviewer 3).

Reviewer 2. Comments for the Author:
I would like to thank the authors for the submission of their revised manuscript. The authors were able to address the majority of the questions I posed, which I feel strengthens the quality of the data and the robustness of the conclusions drawn. While there are some revisions I requested that were not able to be completed due to the availability of the biological material, I do not feel their absence significantly detracts from the overall conclusions of the study. I am happy to recommend this article for publication in Development.

We are grateful to the reviewer for their positive assessment of our work and we would like to thank them for their inputs which have helped us to improve the manuscript.

Reviewer 3. Comments for the Author:

There is a clear phenotype in the absence of six1, which appears to validate previous findings. This is also associated with some changes in SABgal staining, and the RNAseq appears to support mild gene-expression changes in some key senescence genes (the fold change is small, but in many cases, is not validated by QPCR, to a significant degree).

Therefore, there are still gaps in the understanding. There is suggested increase of p21 transcript at E10.5, but no protein shown at this time. P21 staining is shown at 11.5, but not associated with SABgal. p21 does seem to be increased at 13.5, but the association with SABgal is not clear. Unfortunately, there is no genetic rescue hinted at from preliminary data, suggesting that p21 is not a key mediator.

While it does appear there is some effect of the senolytic drug Navitoclax in the ex vivo cultures, that this is specific for an effect on senescence is not clear. No effect on senescence genes was found/shown, and no evidence for increased cell death in senescent cells is provided.

The inverse approach of inducing senescence with Palbociclib, a known senescence mediator, is also interesting. But this more so supports that a drug that induces senescence in the embryo might cause developmental patterning defects, rather than supporting key roles for senescence caused by Six1 deficiency. Overall, this study would need additional functional results to convincingly support that the phenotypes are caused by alterations in senescence.

We are grateful to the reviewer for their comments and inputs which have helped us to improve the manuscript.

We acknowledge that, due to variety of factors, we may not have been able to address all the issues raised in the time allowed for revision. Accordingly, we agree that there may still be still

© 2023. Published by The Company of Biologists under the terms of the Creative Commons Attribution License (https://creativecommons.org/licenses/by/4.0/).

gaps in the understanding of the link between developmental senescence and congenital disorders described in the current manuscript.

In particular, we agree with the reviewer that identifying the key senescence effector involved in these phenotypes is an important issue. At this stage, we consider that the data on genetic rescue using Cdkn1a- or Cdkn2a-null mice is too preliminary to reach conclusions about the involvement of these senescence mediators. Previous related work has shown the relatively limited effects of this kind of genetic rescue experiments on similar phenotypes related to developmental senescence, most likely due to strong compensatory mechanisms (see Muñoz-Espín, Cell, 2013, Storer, Cell, 2013 or Rhinn, PLOS Biol, 2022). Accordingly, we believe that a more extensive analysis with larger sample size would be necessary to obtain a clear answer to this question.

To address the reviewers' and editor's comments in this regard, we have added new text in the Discussion section to acknowledge the limitations of the current study and to describe the scope of follow-up work necessary to tackle the outstanding issues. We hope the edited text now will provide the reader with a more balanced description of both the strengths and limitations of our study.

Third decision letter

MS ID#: DEVELOP/2022/200903

MS TITLE: Dysfunction of programmed embryo senescence is linked to genetic developmental defects.

AUTHORS: Cristina De Lope, Rebeca Garcia-Lucena, Marta Magariños, Yolanda Leon, Nuria Casa-Rodriguez, Nuria Contreras, Carmen Escudero-Iriarte, Isabel Varela-Nieto, Pascal Maire, and Ignacio Palmero
ARTICLE TYPE: Research Report

I am happy to tell you that your manuscript has been accepted for publication in Development, pending our standard ethics checks.

© 2023. Published by The Company of Biologists under the terms of the Creative Commons Attribution License (https://creativecommons.org/licenses/by/4.0/).
